# Charge Transport in LDPE Nanocomposites Part II—Computational Approach

**DOI:** 10.3390/polym8040103

**Published:** 2016-03-23

**Authors:** Anh T. Hoang, Yuriy V. Serdyuk, Stanislaw M. Gubanski

**Affiliations:** Division of High Voltage Engineering, Department of Materials and Manufacturing Technology, Chalmers University of Technology, Gothenburg SE-41296, Sweden; anh.hoang@chalmers.se (A.T.H.); yuriy.serdyuk@chalmers.se (Y.V.S.)

**Keywords:** low-density polyethylene, nanocomposites, charge transport, dc conductivity, charge injection, trapping, de-trapping, charge carrier mobility

## Abstract

A bipolar charge transport model is employed to investigate the remarkable reduction in dc conductivity of low-density polyethylene (LDPE) based material filled with uncoated nanofillers (reported in the first part of this work). The effect of temperature on charge transport is considered and the model outcomes are compared with measured conduction currents. The simulations reveal that the contribution of charge carrier recombination to the total transport process becomes more significant at elevated temperatures. Among the effects caused by the presence of nanoparticles, a reduced charge injection at electrodes has been found as the most essential one. Possible mechanisms for charge injection at different temperatures are therefore discussed.

## 1. Introduction

Accumulation of space charges in polymeric insulation is the main concern during the operation of high voltage direct current (HVDC) cables. It may greatly enhance the electric field inside the insulation bulk that eventually has a detrimental effect on the life expectancy of cables. Thus, knowledge of the generation and transport of charge carriers plays an important role in designing reliable insulation systems of HVDC cable working at high electric field strength. Nowadays, various experimental techniques are used to assess charge dynamics in insulating materials. Apart from that, computer simulations have become popular since they offer great flexibility in investigating effects of different factors and in conducting parametric studies.

A pioneering computer model considering transient processes of charge generation and transport in cable insulation exposed to dc stresses was published in 1994 by Alison and Hill [1] with the aim of reproducing space charge accumulation attained experimentally [2] on a 2.5 mm thick sample of cross-linked polyethylene (XLPE). The model incorporated charge generation due to injection at insulation-electrode interfaces and its transport through material bulk associated with trapping and recombination. Since that time, a variety of models [3,4,5,6,7,8,9,10,11] were developed for studying different physical processes taking place in polyethylene (PE) under a high dc electric field. Le Roy *et al.* [6] proposed a model accounting for de-trapping of charges from deep trapping sites (in contrast to earlier works [1,3,4]) that yielded consistent prediction of experimental results on space charge distribution, conduction currents, and electroluminescence in low-density polyethylene (LDPE). Furthermore, Boufayed *et al.* [7] introduced more realistic exponential distribution of traps instead of two single trap levels (shallow and deep traps) utilized in other models [1,3,5,6]. Most recently, a contribution of surface states at the interfaces between dielectric and electrodes to the dynamics of space charges in LDPE films has been considered [10]. Additionally, formation of charge packets in PE arising as the applied electric field exceeds 100 kV/mm has been studied in [4,11].

It is worth noting that most of the reported simulations were performed for ambient temperatures ~20 °C, which is not the working condition of cable insulation in reality. The actual operating temperatures may reach ~70–80 °C. Moreover, a temperature gradient across the insulation may exist, which affects local characteristics of the material relevant to charge transport. These facts raise questions on the applicability of the existing models for predicting the behavior of insulation in practical situations. This difficulty, in fact, has been dealt with in [9], where the distribution of the electric field and space charges in a cable working under isothermal and temperature gradient conditions were modelled by assuming dependencies of charge carriers’ mobility on temperature and electric field while setting all other model parameters the same as in [6]. The simulated results, however, have not been confirmed by respective experimental data yet.

As for simulations of charge transport in nanocomposites, information about such studies is rather limited. To formulate a consistent model for this case, the basic model of charge transport in pure polymers needs to be extended to account for formation of traps associated with nanofiller particles. These trapping sites may stimulate specific processes which are not present in pure materials. In particular, formation of deep traps that capture mobile carriers injected from electrodes, thus preventing further generation of charges at electrode-material interfaces has been introduced in [12]. Computer simulations utilizing parametric studies to examine the hypotheses as well as to compare contributions of different processes have been reported in [13,14]. The general requirement to such models is that they should be capable to explain the facts that the addition of nanofillers into polymers leads to greatly reduced material dc conductivity [15,16,17] and significantly limited accumulation of space charges [16,18,19] (that actually make, e.g., PE nanocomposites favorable materials for HVDC cable insulation). As for today, such a consistent model of charge transport in nanocomposites that can reproduce experimental results is still lacking and, hence, it needs to be developed.

In the present paper, we study charge transport in LDPE with and without nanofillers at different temperatures by computer simulations using COMSOL Multiphysics (COMSOL AB: Stockholm, Sweden). As a number of processes, *i.e.*, charge generation and transport as well as charge trapping, de-trapping, and recombination have to be accounted for, numerous parameters are involved in the model. It is a common practice to derive these parameters by using fitting procedures that result in additional uncertainties in the model. For avoiding this, we use values of parameters, in particular, the mobility of charges derived from experimental data presented in the first part of this work [17]. The validation of the developed model is examined by comparing the simulated results with conduction currents measured for unfilled LDPE and LDPE/Al_2_O_3_ nanocomposite.

## 2. Model of Charge Transport in Insulating Polymers

### 2.1. The Model

Following the experimental conditions of [17], we consider a flat sample of insulating material of thickness *L* that is sandwiched between a semiconducting anode and a stainless steel cathode. A positive dc voltage *V*_0_ is applied to the anode at zero time, while the cathode is grounded. Since the radii of the electrodes are much larger than the thickness of material sample, the edge effect can be neglected. Thus, the study of charge transport in the flat sample can be reduced to a one-dimensional domain. In such a case, most of the parameters described below are functions of coordinate *x* along insulation thickness and time *t* (note that these dependencies are usually omitted in mathematical expressions below).

To describe conduction process under given conditions, bipolar charge transport model [1,6] is employed. In the model, charge carriers in the material appear due to injection of holes at the anode and electrons at the cathode. The injected charge carriers drift through the material bulk due to the electric field and their transfer is affected by two types of localized states, namely, shallow and deep traps. The former trap type is attributed to structural defects in materials, such as folds, kinks, or ends of polymeric chains. The latter trap type has chemical origin due to the presence of reactive groups such as carbonyl (–C=O), carboxyl (–COOH), *etc.* Charge carriers transported between shallow traps (by hopping) are referred as mobile electrons and holes, whereas the ones being captured in the deep trapping centers are referred as trapped electrons and holes. The trapped carriers occupy traps for a certain residence time, which is considerably longer than that the mobile carriers spend in shallow traps. Charged species captured in deep traps can be released back to the transport state through a de-trapping process. Furthermore, the drift of charge carriers through the material is also associated with their irreversible losses due to various types of recombination. The mathematical description of the model is provided below. Note that model parameters and other quantities in the following equations related to mobile electrons and holes and trapped electrons and holes are denoted by subscripts *e* and *h*, *etr* and *htr*, respectively.

As mentioned, charge carriers can be injected into the insulation through both electrodes as a high dc electrical field is applied. By assuming Schottky’s mechanism, the densities of injected currents can be expressed as:

(1) $$j_{e}(0,t)=AT^{2}\exp\left(-\frac{q(\phi_{K}-\Delta \phi_{K})}{kT}\right)$$

(2) $$j_{h}(L,t)=AT^{2}\exp\left(-\frac{q(\phi_{A}-\Delta \phi_{A})}{kT}\right)$$

Here, the coordinates of the cathode and anode are respectively 0 and *L*, m; *A* is Richardson’s constant (*A* = 1.2 × 10^6^ A∙m^−2^∙K^−2^); *T* being absolute temperature, K; *q* is elementary charge (*q* = 1.6 × 10^−19^ C); φ_A_ and φ_K_ are respectively the barrier heights for charge injection from the anode and cathode, eV; *k* stands for Boltzmann’s constant (*k* = 1.38 × 10^−23^ J·K^−1^). In Equations (1) and (2), Δφ_A,K_ denote the field-lowered barrier heights for charge injection due to electric field *E*_A,K_ at corresponding electrodes:

(3) $$\Delta \phi_{A,K}=\sqrt{\frac{qE_{A,K}}{4\pi \varepsilon_{0}\varepsilon_{r}}}$$

where ε_0_ = 8.854 × 10^−12^ F∙m^−1^ is the permittivity of vacuum and ε*_r_* being material relative permittivity.

Transport of injected charge carriers through insulating materials is governed by a system constituting transport equation (4), current continuity equation (5), differential equation (6), and Poisson’s equation (7):

(4) $$j_{e,h}(x,t)=q\mu_{e,h}n_{e,h}(x,t)E(x,t)$$

(5) $$\frac{\partial n_{e,h}(x,t)}{\partial t}+\frac{1}{q}\frac{\partial }{\partial x}\left(j_{e,h}(x,t)\right)=S_{e,h}(x,t)$$

(6) $$\frac{dn_{etr,htr}(x,t)}{dt}=S_{etr,htr}(x,t)$$

(7) $$\nabla \left(\varepsilon_{0}\varepsilon_{r}E(x,t)\right)=\rho \left(x,t\right)$$

Note that in the transport equation (4) only the drift current is presented, whereas the diffusion current caused by a non-zero gradient of charge densities is neglected. The contribution of diffusion to conduction process has been revealed to be insignificant through additional simulations performed in models with and without considering it. For sake of clarity, the results obtained in these calculations are not presented here. The source terms on the right hand sides of Equations (5) and (6) are introduced below. The term ρ(*x*,*t*) on the right hand side of Equation (7) denotes the total space charge density:

(8) $$\rho =q\left(n_{h}+n_{htr}-n_{e}-n_{etr}\right)$$

As mentioned, the conduction current through the material bulk is due to drift of the injected carriers associated with hopping between shallow traps. To introduce this mechanism, the apparent effective mobilities µ_e,h_ in Equation (4) are defined by the depth of shallow traps φ_e,h_:

(9) $$\mu_{e,h}=\mu_{b(e,h)}\exp\left(-\frac{\phi_{e,h}}{kT}\right)$$

where µ_b(e,h)_ are band mobilities of the respective carriers.

The intensity of trapping process is characterized by trapping coefficients *t*_e,h_ which reflect the probability of capturing of charged species per unit of time. The total trapping rates are quantified as:

(10) $$T_{e,h}=t_{e,h}n_{e,h}\left(1-\frac{n_{etr,htr}}{N_{etr,htr}}\right)$$

where *n*_e,h_ and *n*_etr,htr_ are the number densities of the charge carriers, *N*_etr,htr_ are the total densities of deep traps in the insulating material.

The rates of de-trapping from deep traps, which are considered as potential wells with barrier heights φ_etr,htr_, are introduced as:

(11) $$DT_{e,h}=\nu n_{etr,htr}\exp\left(-\frac{\phi_{etr,htr}}{kT}\right)\frac{n_{etr,htr}}{N_{etr,htr}}$$

where *ν* = *kT*/*h* [7] being the attempt-to-escape frequency and *h* indicating Planck’s constant. The dynamics of filling/releasing traps are described by Equation (6).

In the model, it is assumed that recombination of charges of opposite polarities is mainly between trapped and mobile charges and takes place at trapping sites acting as recombination centers. Probability of recombination between two types of mobile charges is significantly lower [1,8] and can be neglected. In general, the recombination processes lead to the loss in quantity of charged species that are expressed using the rates:

(12) $$\begin{array}{l} R_{eh}=r_{eh}n_{e}n_{h}\\ R_{etrh}=r_{etrh}n_{etr}n_{h}\\ R_{ehtr}=r_{ehtr}n_{e}n_{htr}\\ R_{etrhtr}=r_{etrhtr}n_{etr}n_{htr} \end{array}$$

Here, *r* indicates recombination coefficient and the subscripts *eh*, *etrh*, *ehtr*, *etrhtr* represent recombination between mobile electrons and mobile holes, trapped electrons and mobile holes, mobile electrons and trapped holes, trapped electrons and trapped holes, respectively.

The total rates of generation and losses of mobile and trapped charges in Equations (5) and (6) above can be expressed as:

(13) $$\begin{array}{l} S_{e}=-R_{eh}-R_{ehtr}-T_{e}+DT_{e}\\ S_{h}=-R_{eh}-R_{etrh}-T_{h}+DT_{h}\\ S_{etr}=-R_{etrh}-R_{etrhtr}+T_{e}-DT_{e}\\ S_{htr}=-R_{ehtr}-R_{etrhtr}+T_{h}-DT_{h} \end{array}$$

Note the signs of the terms in Equations (13) which indicate generation (positive rate) or loss (negative rate) mechanisms. Thus, the mobile charges are lost through trapping and recombination and are gained through de-trapping while the latter is the sink of trapped charges.

Finally, the total measurable time-dependent conduction current density through the sample summarizing the contributions from both types of charge carriers is found as:

(14) $$J_{cond}(t)=\frac{1}{L}\int_{0}^{L}\left[j_{h}(x,t)+j_{e}(x,t)\right]\;dx$$

It is well-known that the measured charging current constitutes two components, namely the displacement and conduction currents. As the polarization is modelled by a constant permittivity, the integration of the displacement current over space leads to a term proportional to the time derivative of the voltage, which vanishes exactly for dc applied voltage. Therefore, Equation (14) gives the total current even in the transient phase. Hence, the simulated current density *J*_cond_ (*t*) is used below for comparison with experimentally obtained current density.

The initial and boundary conditions are described as follows. Since a high electric field (32.5 kV/mm) was applied to the insulation in the experiments, the density of charge carriers in the material corresponding to thermal equilibrium prior to voltage application is assumed to be insignificant as compared to the density of injected carriers. Therefore, the densities *n*_e,h_ and *n*_etr,htr_ are set to zero at *t* = 0. Additionally, if charge carriers of certain polarity reach the counter electrode, no extraction barrier for their ejection is specified in the model. In other words, all electrons arriving to the anode and holes arriving to the cathode disappear from the insulation domain. For this, the outward current densities at corresponding electrodes (*i.e*., *j*_e_ at the anode and *j*_h_ at the cathode) are determined in accordance to Equation (4).

### 2.2. Computer Implementation

The presented model was utilized for simulations of charge transport in 80 µm thick films of LDPE with and without nanofillers under conditions corresponding to the experiments [17]. It was implemented in finite element software COMSOL Multiphysics. Numerical solutions of the equation system (5)–(7) were obtained in one-dimensional computational domain. Suitable application modes provided in the software were selected for solving the continuity equation (5), ordinary differential equation (6), and Poisson’s equation (7). The external conduction current density through the sample was calculated by substituting the transport equation (4) into Equation (14). A non-uniform mesh was created with extremely small elements in the vicinity of both electrodes (the smallest element size 0.1 µm), whereas coarser mesh was kept in the middle of the sample. The coupling of different application modes was implemented so that the densities of charge carriers obtained as solutions of Equations (5) and (6) at every time step were updated in Equation (8) for gaining the total space charge density. This was further utilized in Poisson’s equation (7) for deriving the electric field distribution.

## 3. Results of the Simulations and Discussion

### 3.1. Charge Transport in LDPE without Nanofillers

The set of model parameters provided an agreement between the computed and measured results is presented in Table 1. The mobility of holes was set close to the values deduced from the measurements [17], while the effective mobility of electrons was approximately one order of magnitude higher than the mobility of holes (as has been found in [20,21]). It should be noted that the potential barrier height at the anode was set to be lower than that at the cathode as the injection of holes from a semiconducting anode was alleviated as compared to the electron injection from a metal cathode [21]. Furthermore, the barrier heights for de-trapping ~1.0 eV were selected in accordance to trap depth level revealed by the results of thermally stimulated currents [22,23] and our calculations [17] based on demarcation energy model. These levels of trap depth are also in agreement with the values used in other numerical model [6]. The trap densities ~10^21^ m^−3^ were set based on the results obtained in [24]. The recombination coefficients were adopted from [6]. Finally, the trapping coefficients were adjusted to achieve the best fit. The commonly accepted relative permittivity ε*_r_* = 2.3 was used for LDPE.

**Figure 1:**
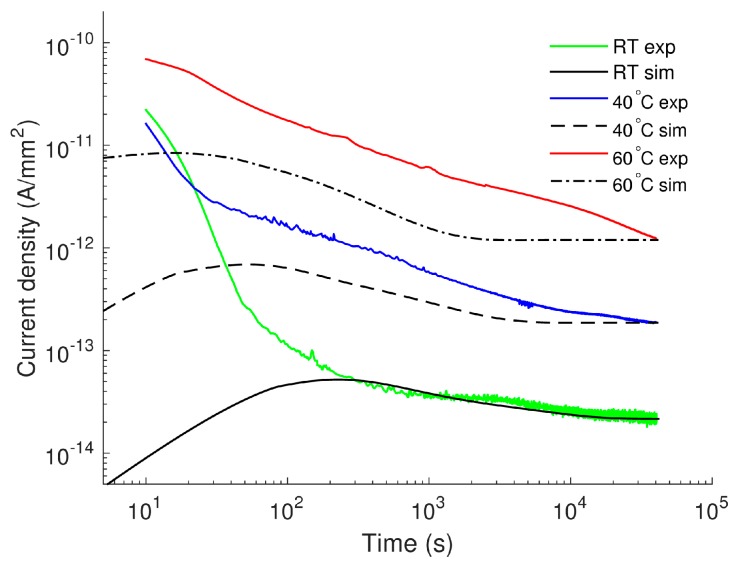
Simulated (referred as “sim” in the legend) and experimental (exp) current densities on LDPE at various temperatures.

The results of the simulations are presented in Figure 1 together with experimental data. Note that the rapid reduction of the measured currents within first 50–70 s particularly prominent at room temperature and 40 °C in Figure 1, is most likely associated with the decaying displacement current arising due to the application of a step voltage. The conduction current component becomes dominating only at longer instants after voltage application (*t* ≥ 10^2^–10^3^ s) and, hence, simulated and experimental currents can be compared only in this stage. As seen in the figure, the current density at room temperature (~22 °C) predicted by the model agrees well with the measured one. At higher temperatures, the computed characteristics are still in line with experimental data but the agreement is getting worse. Broad maxima appear in the simulated currents and the peaks are shifted to a shorter time as the temperature rises.

**Figure 2:**
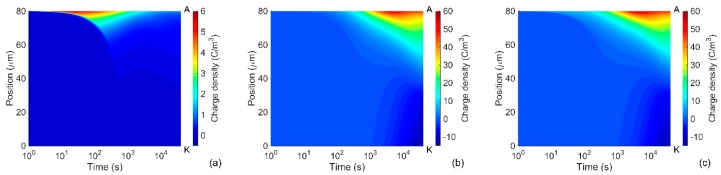
Computed charge density distributions in LDPE at room temperature: (**a**) mobile charges; (**b**) trapped charges; and (**c**) total space charges. Positions of the anode and cathode are indicated by letters A and K, respectively.

**Figure 3:**
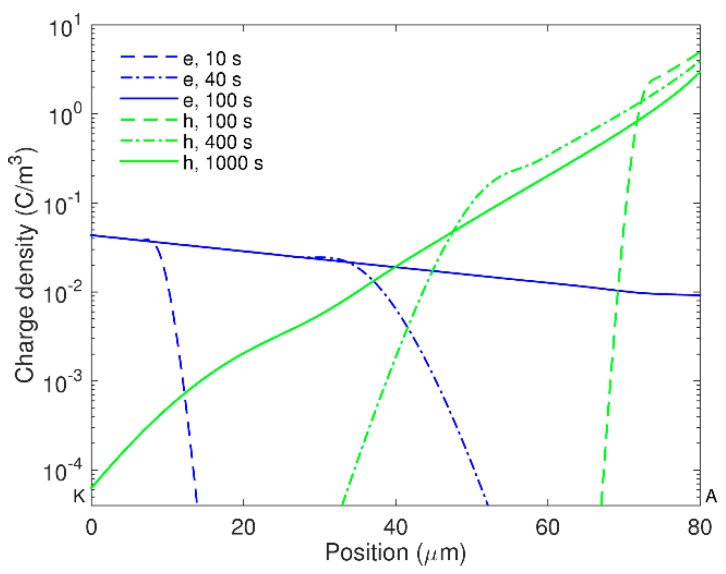
Density profiles of mobile electrons (e) and holes (h) in LDPE at room temperature computed at indicated time.

Distributions of space charges in the material bulk obtained from the simulations at room temperature are illustrated in Figure 2. As seen, the positive carriers dominated over the negative ones throughout the simulated time interval. The dynamics of the space charges in the material can be characterized by their transit times determined as the time duration required for charges traversing through the insulation bulk. The arrival of holes to the cathode and electrons to the anode can be traced by using the profiles of mobile charge density shown in Figure 3. Based on that, the transit time ~100 s for electrons and ~1000 s for holes can be identified that is consistent with the higher (almost one order of magnitude) mobility of electrons as indicated in Table 1. Additionally, the obtained transit times of charge carriers are very close to values calculated as *t*_tr_ = *L*^2^/(µ*V*_0_) assuming insignificant accumulation of space charges in the bulk. As it is observed in Figure 2c, the latter is true for the time shorter than the transit time.

During the transport of injected holes towards the cathode, their density reduces remarkably due to the trapping process and most of the mobile carriers concentrate within a thin layer (5–10 µm) at the vicinity of the anode (Figure 2a). The accumulation of mobile positive carriers in the bulk takes place mostly within first 100 s; thereafter, a reduction in their density can be observed. As seen in Figure 2b, immobile positive charges are gradually built up in the vicinity of the anode within the time interval 10^2^–10^3^ s and its density becomes much higher after 10^3^ s. Thus, the immobile charges are strongly dominating over the mobile ones in the material bulk. The dynamics of positive charge accumulation are controlled by charge injection before 10^2^ s and by charge trapping after 10^3^ s, while a transition process takes place in the time interval 10^2^–10^3^ s. A similar tendency is also observed for the negative charges. In particular, the onset of negative charge accumulation in trapping sites close to the cathode is observed at ~10^3^ s, which results in a considerable amount of trapped electrons in the bulk after 10^4^ s. The variation in the density of the mobile charges leads to the corresponding changes in the simulated conduction current and, hence, a broad maximum appears at time 200–300 s. It should be emphasized that the contribution of mobile electrons to the conduction current cannot be neglected in spite of their remarkably lower density as compared to that of mobile holes. This is because of the higher mobility of electrons than that of holes.

The distribution and evolution of space charges in LDPE at elevated temperatures are not shown here as the main features presented above are preserved. However, one should mention three distinctions, namely, (a) higher levels of charge densities owing to a larger amount of charges injected at elevated temperatures; (b) faster charge transport processes as charge carriers become more mobile with increasing temperature; and (c) the saturation in the computed conduction currents (see Figure 1) observed at ~10^4^ s for 40 °C and at 2 × 10^3^ s for 60 °C. The last feature is not observed within the considered time interval (up to 4 × 10^4^ s) in the simulation at room temperature.

### 3.2. Charge Transport in LDPE Nanocomposites

In this section, charge transport in LDPE filled with 3 wt % of Al_2_O_3_ and MgO nanoparticles is studied. As it is indicated [17], the Al_2_O_3_ particles have spherical shape with an average diameter of 40 nm, whereas the MgO nanoparticles are in rounded hexagonal shape with an average size of 66 nm and a thickness of 10–20 nm. Since the respective volume fractions of the nanofillers are low (0.7–0.8 vol %), the model used in Section 3.1 can also be employed for heterogeneous materials taking into account the effective medium approximations of properties of the composites. Since the experimental results are very close for both nanocomposites under consideration [17], current densities obtained on LDPE/Al_2_O_3_ 3 wt % are employed for comparison with the simulated ones.

#### 3.2.1. Model Parameterization

As it is shown in the first part of the work [17], the measured dc conductivity is substantially lower for LDPE filled with 3 wt % of nanofillers as compared to the unfilled LDPE that is believed to be associated with the weaken charge transport in the nanofilled materials. By recalling the well-known expression for dc conductivity σ = *q*∑*n*_i_ µ_i_, (where *n*_i_ and µ_i_ respectively stand for the density and mobility of *i^th^* type of charge carriers participating in the transport), the reduction in dc conductivity of nanocomposites can be quantitatively related to the decrease in the density of charge carriers and/or the effective mobility.

Despite the density of mobile charge carriers cannot be monitored separately from trapped carriers in space charge measurements, the concentration of mobile charges is anticipated to be lower in nanocomposites. Significant suppression of space charge accumulation in PE nanocomposites observed in various works [13,18,19] has been interpreted by presence of deep traps. Takada *et al.* [12] explained the origin of the deep traps as potential wells induced at the surface of nanoparticles. The depth of potential wells increases strongly with the applied electric field and the dielectric permittivity of the fillers. Thus for LDPE/MgO nanocomposite, the trap depth may be 1–5 eV with the highest level corresponding to the applied field strength of ~200 kV/mm. Further, the trap depth of ~2 eV has been detected in LDPE/MgO nanocomposite by analyzing results of thermally stimulated currents and the origin of these deep traps have been explained by the effect of nanofillers [25]. Based on these findings, the trap depth should be set higher than that for unfilled LDPE. In addition, increased concentrations of traps has been found in nanofilled PE [24]. Such modifications are expected to enhance capturing of charge carriers injected from the electrodes that may result in thinner layers of homocharges in the vicinity of the electrodes as compared to the case of pure material. These, in turn, may reduce the field strength at the interfaces and so decrease the injected currents [24]. This phenomenon can be identified as a screening effect produced by accumulated homocharges. According to the analysis [13], the screening effect yields a higher barrier height for charge injection at electrodes in case of PE-based nanocomposites as compared to the unfilled counterpart. Considering these modifications in material properties brought about by nanofillers, the barrier heights for charge injection at both electrodes were increased by up to 0.1 eV and the density of deep traps rose in five times for the nanocomposites as compared to the reference LDPE (see Table 1). As regards the mobility of charge carriers in nanocomposites, reduced values have been found experimentally [17], which can be elucidated by the alternation in the amorphous region of PE by nanoparticles [26].

According to results of dielectric spectroscopy measurements [27] conducted on LDPE and LDPE/Al_2_O_3_ 3 wt % nanocomposite in frequency range 10^−4^–10^3^ Hz and at three temperatures considered in the model, the relative permittivity was slightly higher (maximum 5%) for the nanocomposite than for reference LDPE. Additionally, the frequency dependencies of the relative permittivity were weak for both materials. The relative permittivity of LDPE nanocomposite was therefore set to 2.3 as for the unfilled LDPE.

#### 3.2.2. Results

**Figure 4:**
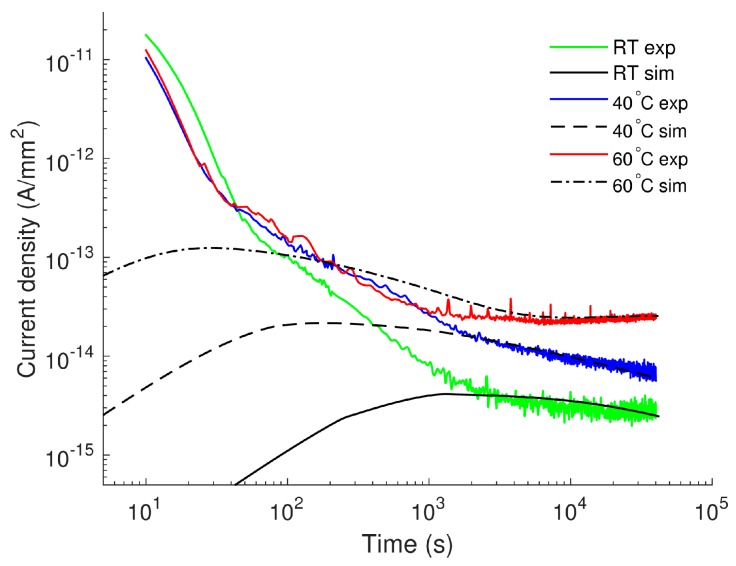
Current densities obtained from simulations (sim) and experiments (exp) on LDPE/Al_2_O_3_ nanocomposite at various temperatures.

The experimental and computed currents in the nanomaterial are compared in Figure 4. Unlike the case of pure LDPE, the conduction current densities predicted by the model show good agreement with the measured ones at all three considered temperatures. At each temperature, the localized peak in the simulated conduction current appears later for the nanocomposite as compared to reference LDPE (Figure 1) due to the lower mobility of charge carriers in nanofilled material.

**Figure 5:**
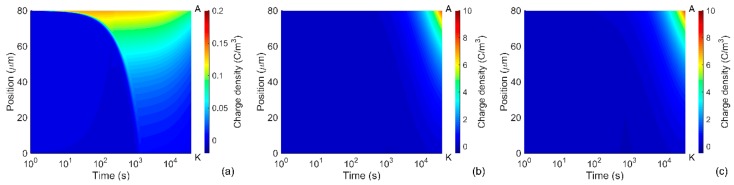
Distributions of charge densities in LDPE nanocomposite obtained from simulations at room temperature: (**a**) mobile charges; (**b**) trapped charges; and (**c**) total space charges. Positions of the anode and cathode are indicated by letters A and K, respectively.

**Figure 6:**
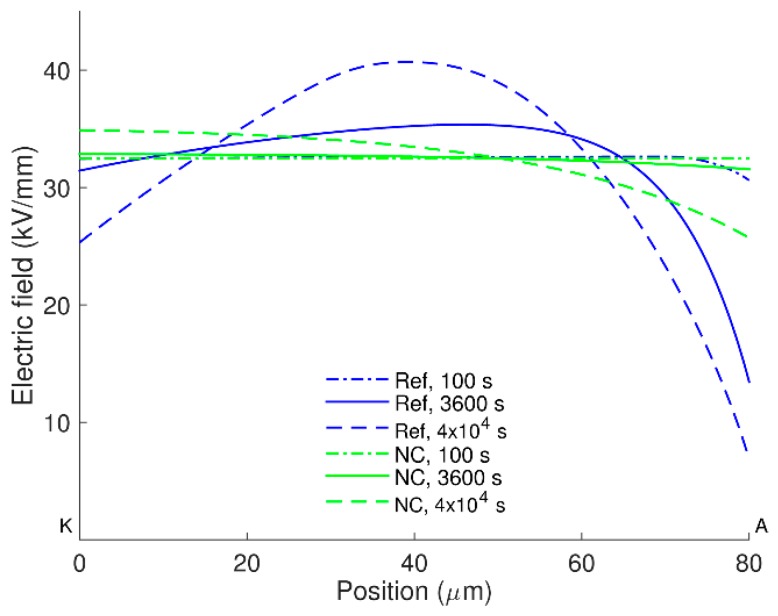
Electric field distributions in LDPE without (Ref) and with (NC) nanofillers at room temperature.

Distributions of charge densities in the bulk of the nanocomposite are shown in Figure 5 for room temperature. Similarly to the reference LDPE, positive charge carriers are dominating in the material and they are mainly concentrated in a thin layer at the vicinity of the anode. As expected, the amount of charges accumulated in the bulk of LDPE nanocomposite is significantly smaller as compared to that in the reference material. Thus, the maximum density of mobile carriers is almost 50 times lower (compare Figure 2a and Figure 5a) while the total space charge density is less than 5 C/m^3^ in most part of the nanocomposite (positions 0–70 µm) and its maximum at the anode is below 9 C/m^3^, Figure 5c. In the unfilled LDPE, the space charges with density exceeding 5 C/m^3^ propagate deeply into the bulk and its maximum is at least six times higher (~55 C/m^3^), see Figure 2c. As a result, the electric field is strongly enhanced inside the reference material, but this is not the case for the nanocomposite. As it is seen in Figure 6, the distortion in electric field distribution in the nanofilled material is negligible at 1 h after voltage application and only small (~7%) field enhancement is observed at the vicinity of the cathode at 4 × 10^4^ s. On the contrary, an appreciable enhancement (~25%) can be noticed in the middle of the sample of the unfilled LDPE at 4 × 10^4^ s.

**Figure 7:**
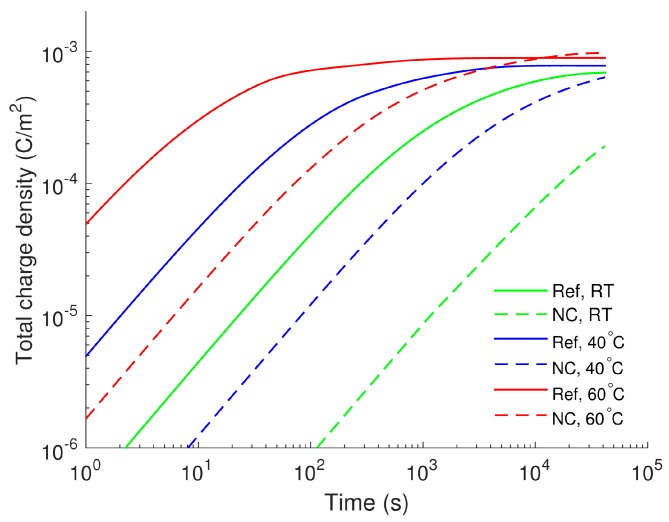
Amount of positive charges accumulated in the bulk of LDPE (Ref) and LDPE nanocomposite (NC).

The quantity of accumulated positive space charges (dominating carriers) calculated as:

(15) $$Q(t)=\int_{0}^{L}q\left[n_{h}\left(x,t\right)+n_{htr}\left(x,t\right)\right]\;dx$$

is presented as a function of time in Figure 7 for all three temperatures. As seen, the amount of charge steadily rises with time and eventually reaches a saturation level ~10^−3^ C/m^2^. The charge magnitudes are lower in the LDPE nanocomposite for all studied temperatures and the differences are more than one order of magnitude in the short time interval, whereas they become smaller at longer time. For simulations at elevated temperatures and time exceeding 10^4^ s, the total positive charges are comparable in both materials.

### 3.3. Influence of Different Physical Processes on Charge Transport

As discussed above, the weakening in charge transport in nanofilled LDPE as compared to the unfilled one can be attributed to the reduced charge injection at electrode-insulation interfaces, to the decreased charge carrier mobility, to the increased probability of charge capturing in and the decrease of charge release from deep traps. However, it is unclear which process among the above-mentioned mainly contributes to the lowering of the conduction in LDPE nanocomposite. In other words, what behavior of insulation is changed most noticeable due to the addition of nanofillers into LDPE?

**Figure 8:**
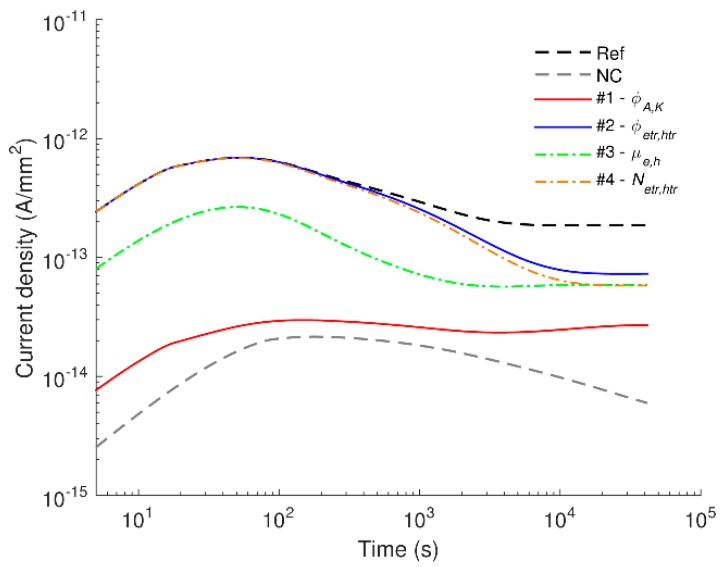
Simulated conduction currents at 40 °C obtained by varying model parameters. Four scenarios are considered as shown in Table 2. The parameters being changed are indicated in the legend.

To address this question, we assume that only one type of parameters incorporated in the model for pure LDPE and associated with a certain physical process is modified at a time to the values used in the model of LDPE nanocomposite (Table 1), while all other parameters are kept unchanged. Thus, four scenarios are considered as described in Table 2 and the obtained results (conduction currents at temperature 40 °C) are illustrated in Figure 8, where the simulated currents in LDPE and its nanocomposite are also shown for comparison. As can be seen, the conduction current drops significantly down to the level close to that in the nanocomposite while increasing the injection barriers alone and less pronounced decline is observed in three other situations. The effect of charge mobility on the conduction current is almost the same in the studied time interval, whereas the influence of trap energy (φ_tr_) and trap density (*N*_tr_) is remarkable only at times exceeding 10^4^ s. Based on the results of the analysis, we found that the decrease in charge injection at the electrodes mainly accounts for the weakening of conduction in LDPE nanocomposite and so for the suppression of space charge build-up in the bulk.

**Figure 9:**
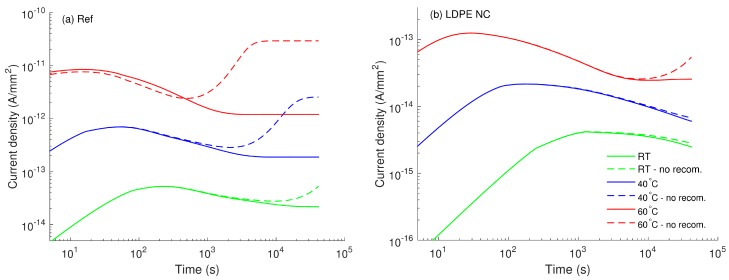
Simulated conduction currents obtained from models with (solid curves) and without (dashed curves) charge recombination.

The contribution of charge recombination to the conduction current is examined by considering charge transport models with and without accounting for this particular process. The simulated conduction currents in both materials are compared in Figure 9. As it is found, charge recombination is essential in pure material and in the nanocomposite at 60 °C. Neglecting this process yields a rapid rise of the simulated currents, especially at elevated temperatures. The marked increase in the conduction currents obtained in the model without recombination is due to the excess of mobile charges in the bulk, which would be neutralized if recombination is included. In this context, it is interesting to observe that such neutralization is not of importance for the nanofilled material at room temperature and at 40 °C. The differences in the simulated outcomes for LDPE with and without nanoparticles can be attributed to the strong distinctions in the amount of charge carriers generated in these materials. The obtained results also indicate that charge recombination cannot be underestimated in the charge transport model at elevated temperatures, even though its contribution is minor at room temperature.

### 3.4. Discussion

As mentioned above, broad maxima are observed in the simulated time variations of the current densities and the time *t*_p_ corresponding to the current peaks is temperature-dependent (see Figure 1 and Figure 4). These localized peaks are not exhibited in our experimental results [17]. Indeed, localized peaks are often detected in time-domain currents measured on oxidized LDPE [28] rather than on the non-oxidized counterpart [29]. Their appearance has been explained by the high concentration of carbonyl groups (–C=O) in the former material as compared to the latter. The carbonyl groups give rise to the hopping transport of mobile charge carriers in the bulk that eventually increases the conduction current [28]. Current maxima are therefore observed as a consequence of the build-up of significant mobile charges in the bulk [30]. In contrast, lower conduction currents are detected for the non-oxidized PE and the peaks are most probably hidden by the displacement current. The latter arises in transient processes activated by the voltage application due to orientation of polar groups existing in PE. In LDPE samples used in this investigation, the presence of antioxidant is anticipated to suppress the formation of carbonyl groups that explains the absence of the current maxima in the measured charging currents. Nevertheless, the current maxima in the simulated characteristics are of interest. According to the analysis by Many and Rakavy [30] for a single-carrier model in trap-free materials, the peak of transient current corresponds to the arrival of charges at the counter electrode. The peak time *t*_p_ can be found as *t*_p_ = 0.787 × *t*_tr_, where *t*_tr_ = *L*^2^/(µ*V*_0_). Unlike the case of trap-free materials, different features are noted in the bipolar charge transport model for materials with traps. As charge trapping strongly reduces the density of mobile carriers, their total density and the current density achieve maxima well before the arrival of the dominating charge carriers at the counter electrode. Thus, in a correlation between *t*_p_ and *t*_tr_ established by using results obtained in Section 3.1 and Section 3.2.2, the multiplication factor should be much lower than 0.787.

Another noteworthy feature is that the accumulation of trapped charges in the unfilled LDPE at elevated temperatures becomes saturated after certain time, e.g., at 2 × 10^3^ s at 60 °C. As the trapped charges constitute the main part of the space charges, the same tendency is observed for the latter, yielding unchanged electric field distribution in the insulation bulk afterward. This eventually causes the steady state of simulated current density as seen in Figure 1. In order to avoid the early saturation in the simulated external current density, the trapping coefficients have been adjusted as increasing with temperature, which can be interpreted as the increased probability of charge trapping due to the presence of an increased amount of charges generated at higher temperatures. However, we realized that the steady state in the current density is persistent for simulations at elevated temperatures and it is unavoidable for the described model of charge transport. In fact, the saturation in the simulated trapped charges has been noted in [5] at 9 × 10^3^ s and the saturated conduction currents are clearly illustrated in [14]. In both cases, the simulations of charge transport were implemented for the conditions of ambient temperature. Note that at room temperature, the steady state in the simulated characteristics is not exhibited within the considered time range in the present study; it only arises at elevated temperatures. The effect of temperature on the saturation of the simulated characteristics could be attributed to the fact that the injected currents described by Schottky’s law, Equations (1) and (2), may not fully reflect the physical processes at the electrodes. According to Schottky’s law, the amount of injected mobile carriers at the electrodes increases substantially with temperature and, hence, the traps in the insulation bulk can be filled more easily at higher temperatures, yielding the premature saturation in the density of trapped charges and so for the simulated conduction currents.

The applicability of Schottky’s mechanism for charge injection at electrode-insulation interfaces is in fact questionable [31]. First of all, the distance *x_max_* from the electrode corresponding to the maximum of potential barrier is too long so that an electron may be thermalized by collisions before reaching the barrier [31]. Secondly, the barrier height for injection used in simulations (~1.1–1.3 eV) is much smaller than that at metal-PE interfaces obtained by using density functional theory (DFT) calculations, e.g., [32]. Taylor and Lewis [33] analyzed currents measured on thin films of polyethylene terephthalate (PET) and PE exposed to a wide range of applied electric field at various temperatures and proposed an alternative to Schottky’s mechanism where a general form followed Equation (16) instead of the coulombic form by Equation (17) of the potential barrier at electrodes is utilized:

(16) $$\phi_{G}(x)=-\frac{Kq}{{\left(ax\right)}^{n}}$$

(17) $$\phi_{C}(x)=-\frac{q^{2}}{16\pi \varepsilon x}$$

In Equation (16), *K*, *a*, and *n* are positive constants, *K* accounts for contributions of the charge *q* and material permittivity ε presented in the coulombic form of Equation (17). In Equations (16) and (17), *x* is the distance from the electrode. The widely used Schottky injection law with the coulombic form of the potential barrier is a special case of the general form when the exponent *n* equals unity. For PET and PE, it has been found that the constant *n* is much lower than unity. The departure from the image-law potential barrier according to Equation (17) has been explained by space charge build-up at the interfaces and in the insulation bulk as well [33]. Additionally, zero-field activation energies derived for PET and PE were respectively 2.58 and 2.14 eV, which were interpreted as the potential barriers of the general law [33]. These values are closer to the results of DFT calculations obtained recently [32] as compared to the commonly used ones in the simulations. It should be mentioned that transient processes due to charge trapping, de-trapping, and recombination in the bulk have not been considered in these analyses [33] and, hence, the proposed approach should be reconsidered by taking into account the bulk processes. This may provide better explanation of experimental data obtained for the reference LDPE at elevated temperatures.

## 4. Conclusions

Charge transport in LDPE and its nanocomposites at different temperatures has been studied by numerical simulations. By achieving a good agreement between simulated and measured conduction currents, we reveal quantitative changes in various physical processes taking place in the insulating materials caused by the presence of nanofillers. In particular, the weakening charge transport in the nanodielectrics as compared to the unfilled LDPE is associated with the increased barrier heights for charge injection at electrodes, the reduced charge mobility, and the increased trap energy and trap density. Simulated results also demonstrate that space charge accumulation and electric field enhancement are less noticeable in LDPE nanocomposites than in the unfilled counterpart. The reduced conduction currents and the suppression of space charge accumulation in nanofilled LDPE are most likely governed by the modification of the barrier heights for charge injection at the dielectric-electrode interfaces. Furthermore, the application of Schottky’s mechanism for describing charge injection at electrodes in the simulation is still questionable as it does not fully explain the thermally activated behavior of the conduction currents obtained experimentally.

## Figures and Tables

**Table 1:** Parameters used in models for fitting the measured conduction currents on LDPE and its nanocomposites at various temperatures. RT stands for room temperature (~22 °C).

parameters	LDPE			LDPE Nanocomposites		
	RT	40 °C	60 °C	RT	40 °C	60 °C
Effective mobility						
µ_e_, m^2^·V^−1^·s^−1^	3.0 × 10^−14^	1.5 × 10^−13^	5.5 × 10^−13^	1.0 × 10^−14^	3.0 × 10^−14^	7.0 × 10^−14^
µ_h_, m^2^·V^−1^·s^−1^	2.5 × 10^−15^	1.2 × 10^−14^	5.0 × 10^−14^	2.0 × 10^−15^	6.0 × 10^−15^	1.4 × 10^−14^
Trapping coefficients						
*t*_e_, s^−1^	0.02	0.08	0.25	0.002	0.022	0.13
*t*_h_, s^−1^	0.01	0.03	0.08	0.002	0.022	0.13
De-trapping barrier height						
φ_etr_, eV	0.93	0.96	1.00	1.00		
φ_htr_, eV	0.93	0.96	1.00	1.00		
Deep trap density						
*N*_etr_, m^−3^	1.25 × 10^21^			6.25 × 10^21^		
*N*_htr_, m^−3^	1.25 × 10^21^			6.25 × 10^21^		
Schottky injection barriers						
φ_K_, eV	1.22			1.31		
φ_A_, eV	1.16			1.26		
Recombination coefficients						
*r*_etrhtr_, m^3^·s^−1^	6.4 × 10^−22^			6.4 × 10^−22^		
*r*_etrh_, m^3^·s^−1^	6.4 × 10^−22^			6.4 × 10^−22^		
*r*_ehtr_, m^3^·s^−1^	6.4 × 10^−22^			6.4 × 10^−22^		
*r*_eh_, m^3^·s^−1^	0			0		
Relative permittivity						
ε_r_	2.3			2.3		

**Table 2:** Scenarios for simulations with varying parameters. Model parameters of each scenario are the same as for simulating charge transport in LDPE, except for those provided in the right column. Charge mobilities (in m^2^·V^−1^·s^−1^) are listed in order of increasing temperature (RT; 40 °C; 60 °C).

Scenario	Description	Model parameters	Modified parameters
#1	Reduction of charge injection at electrodes	Charge injection barrier heights as for the nanocomposite, all other parameters as for LDPE	φ_K_ = 1.31 eV φ_A_ = 1.26 eV
#2	Reduction of charges released from deep traps	De-trapping barrier heights as for the nanocomposite, all other parameters as for LDPE	φ_etr_ = 1.00 eV φ_htr_ = 1.00 eV
#3	Reduction of charge carrier mobility	Mobility of electrons and holes as for the nanocomposite, all other parameters as for LDPE	µ_e_ = 1 × 10^−14^; 3 × 10^−14^; 7 × 10^−14^ µ_h_ = 2 × 10^−15^; 6 × 10^−15^; 1.4 × 10^−14^
#4	Increase of trap densities	Trap densities as for the nanocomposite, all other parameters as for LDPE	*N*_etr_ = 6.25 × 10^21^ m^−3^ *N*_htr_ = 6.25 × 10^21^ m^−3^
